# Heme Oxygenase-1 Induction and Organic Nitrate Therapy: Beneficial Effects on Endothelial Dysfunction, Nitrate Tolerance, and Vascular Oxidative Stress

**DOI:** 10.1155/2012/842632

**Published:** 2012-03-07

**Authors:** Andreas Daiber, Matthias Oelze, Philip Wenzel, Franziska Bollmann, Andrea Pautz, Hartmut Kleinert

**Affiliations:** ^1^2nd Medical Clinic, Department of Cardiology, University Medical Center of the Johannes Gutenberg University, 55131 Mainz, Germany; ^2^The Center for Thrombosis and Hemostasis, University Medical Center of the Johannes Gutenberg University, 55131 Mainz, Germany; ^3^Department of Pharmacology, University Medical Center of the Johannes Gutenberg University, 55131 Mainz, Germany

## Abstract

Organic nitrates are a group of very effective anti-ischemic drugs. They are used for the treatment of patients with stable angina, acute myocardial infarction, and chronic congestive heart failure. A major therapeutic limitation inherent to organic nitrates is the development of tolerance, which occurs during chronic treatment with these agents, and this phenomenon is largely based on induction of oxidative stress with subsequent endothelial dysfunction. We therefore speculated that induction of heme oxygenase-1 (HO-1) could be an efficient strategy to overcome nitrate tolerance and the associated side effects. Indeed, we found that hemin cotreatment prevented the development of nitrate tolerance and vascular oxidative stress in response to chronic nitroglycerin therapy. Vice versa, pentaerithrityl tetranitrate (PETN), a nitrate that was previously reported to be devoid of adverse side effects, displayed tolerance and oxidative stress when the HO-1 pathway was blocked pharmacologically or genetically by using HO-1^+/–^ mice. Recently, we identified activation of Nrf2 and HuR as a principle mechanism of HO-1 induction by PETN. With the present paper, we present and discuss our recent and previous findings on the role of HO-1 for the prevention of nitroglycerin-induced nitrate tolerance and for the beneficial effects of PETN therapy.

## 1. Organic Nitrate Therapy and Side Effects

Nitroglycerin (GTN) has been one of the most widely used anti-ischemic drugs for more than a century. Given acutely, organic nitrates are excellent agents for the treatment of stable effort angina, acute myocardial infarction, chronic congestive heart failure, pulmonary edema, and severe arterial hypertension (for review see [1, 2]). The chronic efficacy of nitrates, however, is blunted due to the development of nitrate tolerance and endothelial dysfunction, phenomena that are largely associated with increased vascular oxidative stress (for review see [1–5]). Oxidative stress was demonstrated to be a hallmark of most cardiovascular diseases [6]. The term oxidative stress defines a state with either increased formation of reactive oxygen and nitrogen species (RONS) and/or impaired cellular antioxidant defense system (e.g., downregulation of important antioxidant proteins) with subsequent depletion of low-molecular-weight antioxidants and a shift in the cellular redox balance. The central role of the endothelium for the regulation of vascular tone makes it a vulnerable target for RONS which can interfere at many positions with the NO/cGMP signaling cascade [7].

It is well established that most organic nitrates cause nitrate tolerance and/or cross-tolerance to endothelium-dependent vasodilators (e.g., acetylcholine) [8–11]. The first report on a role for oxidative stress in the development of nitrate tolerance was published in 1995 by Münzel and coworkers for nitroglycerin therapy [12]. These authors found that superoxide levels were twofold higher in aortic segments from nitrate tolerant vessels with intact endothelium. Based on these findings, they suspected that the enhanced levels of superoxide in nitroglycerin tolerant vessels might contribute not only to nitroglycerin tolerance, but also to cross-tolerance to 3-morpholinosydnonimine (Sin-1) and endogenous NO production stimulated by acetylcholine. To test this hypothesis, they examined the effects of bovine Cu, Zn-superoxide dismutase (SOD) entrapped in pH sensitive liposomes. In nitroglycerin-tolerant aortic segments with endothelium, liposomal SOD markedly enhanced the relaxations evoked by nitroglycerin, Sin-1, and acetylcholine. The source of RONS formation in the setting of nitrate tolerance was first found to be NADH oxidase. This finding was mainly based on the observation that the superoxide signal was most pronounced in the presence of NADH and that it was located in the particulate and not cytosolic fraction [13]. More compelling data came from the observation that the protein kinase C inhibition effectively suppressed nitroglycerin-induced vascular RONS formation and vasoconstrictor supersensitivity in tolerant vessels, keeping in mind that protein kinase C activates NADPH oxidase [14, 15].

Since nitroglycerin is thought to release NO and induce superoxide formation simultaneously, the formation of peroxynitrite from the reaction of NO and superoxide could be expected. Indeed, some studies have reported on increased levels of tyrosine-nitrated proteins, which is a marker for increased peroxynitrite formation in tissue from nitrate-tolerant animals [16]. We could also identify higher concentrations of nitrated prostacyclin synthase and decreased prostacyclin levels in these animals [17]. Indirect proof for a role of peroxynitrite for nitrate tolerance came from the observation that hydralazine, which efficiently improves nitrate tolerance, is a powerful peroxynitrite scavenger and inhibitor of protein tyrosine nitration [18]. Moreover, authentic or in situ generated (Sin-1-derived) peroxynitrite was most efficient in inhibiting the bioactivating enzyme of nitroglycerin [19]. In addition, three independent reports provided data that peroxynitrite plays a central role in the development and pathogenesis of nitrate tolerance [20–22].

The concept of NAD(P)H oxidase-driven RONS formation as the most important source of oxidative stress in nitrate tolerance was accepted for almost 10 years. In 2004, we reported for the first time on mitochondrial ROS formation in nitroglycerin induced tolerance [23], although bioactivation of nitroglycerin by mitochondrial aldehyde dehydrogenase (ALDH-2) was already reported 2 years earlier [24]. Despite the fact that the harmful effects of organic nitrates on mitochondria have already been described in the 1960s by Needleman and coworkers (mitochondrial swelling, thiol depletion, and impaired respiration) [25, 26], it took more than 40 years to reveal the pivotal role of mitochondria in nitroglycerin toxicity [23, 24, 27]. To test this hypothesis, we used mice with heterozygous Mn-SOD deficiency (Mn-SOD^+/−^), which is the mitochondrial isoform of superoxide dismutases [28, 29]. Nitroglycerin-driven vascular and mitochondrial ROS formation was increased in Mn-SOD^+/−^ mice, and, vice versa, the ALDH-2 activity in these samples was decreased by nitroglycerin in a more pronounced manner. Moreover, nitroglycerin potency was significantly impaired in response to low-dose nitroglycerin *in vivo* treatment indicating the development of nitrate tolerance by this low dose in Mn-SOD^+/−^ mice but not in wild-type controls. The detrimental role of mitochondrial RONS formation for the development of GTN-induced nitrate tolerance was further supported by a subsequent report on the prevention of GTN side effects by the mitochondria-targeted antioxidant mitoquinone (mitoQ) [30].

## 2. Effects of HO-1 Induction and Suppression on Nitrate Tolerance and Oxidative Stress

**Figure 1:**
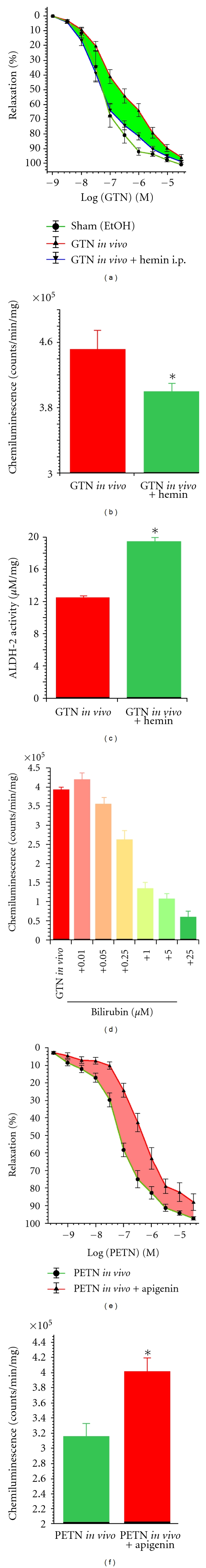
Effects of the HO-1 inducer hemin on GTN-induced tolerance and effects of the HO-1 suppressor apigenin on PETN side effects. Hemin (25 mg/kg) was administrated by single i.p. injection on day 3 of GTN treatment (6.6 *μ*g/kg/min for 4 days via s.c. infusion) and markedly improved vascular GTN responsiveness (see area between curves) as demonstrated by isometric tension studies (a), a significant decrease in mitochondrial ROS formation (b), and improvement of mitochondrial ALDH-2 activity (c). Effects of bolus bilirubin on ROS formation in isolated heart mitochondria from GTN *in vivo *treated rats were determined by L-012 (100 *μ*M) ECL in the presence of 2.5 mM succinate and bilirubin (0–25 *μ*M) (d). Apigenin (10 mg/kg/d) was coinfused over 4d together with PETN (10.5 *μ*g/kg/min for 4 days via s.c. infusion). Apigenin cotreatment decreased PETN vasodilator potency (see area between curves) and induced a tolerance-like right shift in the PETN concentration-relaxation-curve (E). This observation was accompanied by increased mitochondrial ROS formation (F). Data are mean ± SEM of *n*  =  8–12 (a), 40 (b), 6–18 (c), 4–6 (d), 9–11 (e), and 28–43 (f) independent experiments. *P < 0.05 versus GTN or PETN treatment. Modified from [31].

With a previous study, we demonstrated that chronic nitroglycerin (GTN) therapy results in impaired vasodilatory potency of GTN (nitrate tolerance) and of the endothelium-dependent vasodilator acetylcholine (endothelial dysfunction) as well as increased vascular and mitochondrial RONS formation [31]. Since another organic nitrate (PETN) was previously described to be devoid of nitrate tolerance and induction of oxidative stress due to induction of the heme oxygenase-1 (HO-1) system [35, 36], we hypothesized that pharmacological activation of the HO-1 system may be suitable to prevent GTN-dependent side effects. In deed, cotreatment of GTN-infused rats with the potent HO-1 inducer hemin completely prevented nitrate tolerance (restored the GTN-dependent relaxation), restored NO/cGMP signaling, increased the activity of the GTN bioactivating enzyme ALDH-2, and suppressed the mitochondrial RONS formation (Figures 1(a)–1(c)) [31]. The HO-1 product bilirubin suppressed GTN-induced RONS formation in isolated heart mitochondria (Figure 1(d)). To test the essential role of HO-1 for the tolerance devoid action of PETN, we cotreated PETN-infused rats with the HO-1 suppressor apigenin and observed a tolerance-like phenomenon displaying impaired PETN-dependent relaxation, disturbed NO/cGMP signaling, and increased mitochondrial RONS formation (Figures 1(e)-1(f)) [31].

These observations are in good accordance with previous reports on HO-1 induction by statins [37, 38] and prevention of nitrate tolerance in GTN-infused experimental animals [39] as well as human individuals [40, 41]. The role of HO-1 for prevention of organic nitrate induced tolerance, endothelial dysfunction, and oxidative stress is further supported by observations that the HO-1 product bilirubin efficiently scavenged GTN-induced RONS (most probably peroxynitrite) formation in isolated mitochondria [29, 31]. Likewise, the HO-1 products bilirubin and carbon monoxide as well as PETN increased the expression of the GTP-cyclohydrolase-1 (GCH-1), the most important enzyme for *de novo* synthesis of tetrahydrobiopterin (BH_4_), an essential cofactor for endothelial NO synthase (eNOS) function [34, 42]. In contrast, GTN *in vivo* therapy decreased the expression of the GCH-1 [43]. Since BH_4_ levels are directly linked to eNOS activity and endothelial function [44, 45] and GCH-1 is oxidatively degraded by activation of the proteasome26S [46], activation of antioxidant pathways by induction of HO-1 may represent an attractive explanation for the tolerance and endothelial dysfunction devoid profile of chronic PETN therapy and the undesired side effects of most other organic nitrates (but most pronounced for GTN treatment). Similar observations were made for extracellular superoxide dismutase (ecSOD), a downstream target of HO-1 and its products [47], which is upregulated by PETN [34, 48] but not by isosorbide-5-mononitrate (ISMN). Moreover, it was recently reported that HO-1 plays a significant role in the maintenance of soluble guanylyl cyclase (sGC) in a reduced heme state providing another important function for HO-1 in the regulatory pathways of vascular tone [49]. This novel beneficial property of HO-1 could also significantly improve vascular dysfunction in the setting of nitrate tolerance. These observations underline the distinct properties of organic nitrates, and these drugs do not represent a class with homogeneous effects [50] but also challenge the traditional assumption that all organic nitrates release the same vasoactive species (nitric oxide)—an assumption that was already challenged for GTN in 2003 [51] (and reviewed in [1, 5]).

 In addition, previous work has demonstrated that organic nitrates have distinct effects on the function and survival of circulating angiogenic cells (formally known as endothelial progenitor cells) [52, 53]. These studies showed that isosorbide dinitrate in contrast to PETN impair the migration and incorporation activities of these circulating angiogenic cells in an experimental model of myocardial infarction, whereas GTN *in vitro* exposure increased apoptosis while decreasing phenotypic differentiation, migration, and mitochondrial dehydrogenase activity in these cells. In a subsequent study, Lin et al. investigated the involvement of heme oxygenase-1 for the related neovascularization process by hematopoietic stem cells and endothelial progenitor cells in the infarcted area [54]. Thum et al. have shown that the impaired function of these circulating angiogenic cells is based on oxidative stress as envisaged in the setting of diabetes, leading to eNOS uncoupling, which was improved by antioxidants (e.g., superoxide dismutase) [55]. These findings underline the importance of maintaining the BH_4_ levels to prevent eNOS uncoupling and the role of HO-1 for this antioxidant mechanism via increase in GCH-1 expression by carbon monoxide and bilirubin as outlined above. This concept is further supported by protective effects of folic acid (a precursor of BH_4_) on impaired endothelial function in GTN-treated healthy volunteers [56], and decreased BH_4_ levels in GTN-treated rabbits were restored by cotherapy with pioglitazone [57]. It should be noted that another group found no association between aortic BH_4_ content and eNOS function in response to GTN *ex vivo* and *in vivo* treatment [58].

Finally, it should be mentioned that oxidative stress in response to organic nitrates may also be protective by a process called ischemic preconditioning (IPC) [59–61]. Recently, the involvement of HO-1 in organic nitrate-mediated IPC was proposed [59, 62] as an explanation for the sustained IPC protective effect under chronic PETN therapy but loss of this beneficial effect under chronic GTN therapy. This is in accordance with the accepted view that HO-1 plays a role in IPC [63, 64].

## 3. Molecular Proof of a Role of HO-1 for the Tolerance-Devoid Profile of PETN by Using HO-1^+/−^ Mice

**Figure 2:**
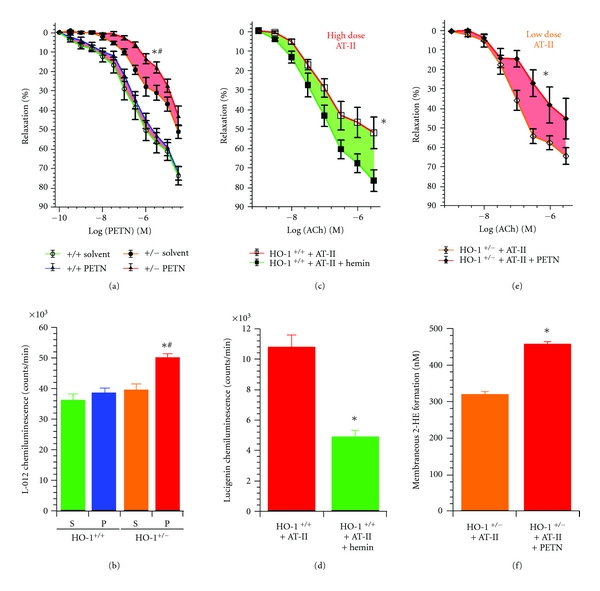
Effects of HO-1 deficiency versus HO-1 induction on vascular improvement by pentaerithrityl tetranitrate (PETN). (a, b) PETN treatment (75 mg/kg/d for 4d) had no effect on PETN potency (PETN-induced relaxation) in aorta from control mice (HO-1^+/+^) but caused nitrate tolerance (see area between curves) in aorta from mice with partial HO-1 deficiency (HO-1^+/−^). In accordance, cardiac mitochondrial ROS formation (L-012 ECL) was increased in PETN-treated HO-1^+/−^ mice. *P* < 0.05: *versus HO-1^+/+^/DMSO; ^#^versus HO-1^+/−^/DMSO. S: solvent; P: PETN-treated. (c, d) Hemin (25 mg/kg i.p.)-triggered HO-1 induction improved high-dose AT-II (1 mg/kg/d for 7d)-induced endothelial dysfunction (ACh-response) in aorta (see area between curves) and NADPH oxidase activity in heart (lucigenin ECL) from control mice (HO-1^+/+^). *P* < 0.05: *versus AT-II-treated HO-1^+/+^/DMSO. (e,  f) PETN (75 mg/kg/d for 7d) failed to prevent endothelial dysfunction (ACh-response) induced by low-dose AT-II (0.1 mg/kg/d for 7d) in aorta from HO-1^+/−^ mice (see area between curves). In accordance, PETN did not improve NADPH oxidase activity (2-HE formation by HPLC analysis) in cardiac samples from AT-II-treated HO-1^+/−^ mice. *P* < 0.05: *versus AT-II-treated HO-1^+/−^/DMSO. All data are mean ± SEM of aortic rings and hearts from 4-5 animals/group. Modified from [32].

The role of HO-1 as the antioxidative principle of PETN was elucidated by 3-key experiments aiming to prove this hypothesis at a molecular basis [32]. The first experimental setup consisted of the treatment of control (HO-1^+/+^) and partially deficient (HO-1^+/−^) mice with PETN. In HO-1^+/−^ but not HO-1^+/+^ mice, PETN infusion induced desensitization to PETN-induced vasorelaxation (envisaged by impaired vasodilator potency of the drug in isolated aortic segments) and increased mitochondrial ROS formation (Figures 2(a) and 2(b)), demonstrating nitrate tolerance to PETN in a setting of HO-1 deficiency. The second approach was on the basis of HO-1 induction by the known inducer of this enzyme, hemin, which improved angiotensin-II-(high-dose) dependent endothelial dysfunction and prevented activation of NADPH oxidase in HO-1^+/+^ mice (Figures 2(c) and 2(d)). The third experiment demonstrated that PETN did not improve endothelial dysfunction and cardiac oxidative stress in angiotensin-II-(low-dose) treated HO-1^+/−^ mice but further impaired vascular function and increased ROS formation in this setting (Figure 2(e) and 2(f)). It is somewhat surprising that already heterozygous deficiency in HO-1 leads to complete loss of the beneficial and protective effects of PETN but may also underline how essential the upregulation of HO-1 is to prevent nitrate tolerance, endothelial dysfunction, and oxidative stress under chronic therapy with organic nitrates. It would be of great clinical importance to study the vasodilatory potency of GTN and development of nitrate tolerance under chronic GTN therapy in human individuals with Morbus Meulengracht (Gilbert's syndrome) to translate our preclinical data from bench to bedside and to further explore the therapeutical potential of HO-1 induction to overcome the side effects of GTN therapy. Since human subjects with hyperbilirubinemia (e.g., new born with mild jaundice or patients with Gilbert's syndrome) have a better prognosis and have a significant lower cardiovascular risk [65], it may be hypothesized that they will also display a lower degree of nitrate tolerance in response to chronic GTN therapy. 

**Figure 3:**
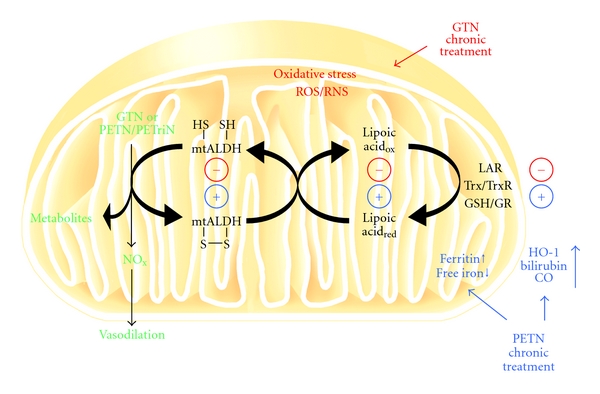
Scheme illustrating the mechanisms underlying the oxidative stress concept of nitrate tolerance in response to GTN treatment and the mechanisms underlying the beneficial vascular effects in response to PETN. PETN and GTN are bioactivated by mitochondrial ALDH (ALDH-2) yielding 1,2-glyceryl dinitrate and PETriN, respectively, as well as a yet undefined nitrogen species (NO*_x_*, probably nitrite) that undergoes further reduction by the mitochondrial respiratory chain or acidic disproportionation to form an activator of sGC (probably nitrico xide). GTN treatment induces mitochondrial reactive oxygen and nitrogen species formation (ROS/RNS). These ROS/RNS in turn inhibit the GTN bioactivation process by inactivation of ALDH-2 or by inhibiting the repair system of the ALDH-2, which includes lipoic acid, as well as a reductase system depending on the NADH or NADPH (lipoicacid reductase (LAR), thioredoxin/thioredoxin reductase (Trx/TrxR) or glutathione/glutathione reductase (GSH/GR). In contrast to GTN, PETN provides potent antioxidative effects by inducing HO-1 and ferritin, which in turn decrease ROS levels and therefore protect the ALDH-2 from ROS mediated inactivation. Adapted from [31].

 The beneficial effects of HO-1 induction on mitochondrial RONS formation may be attributed to the presence of HO-1 in mitochondria [66] and the improvement of mitochondrial biogenesis as well as suppression of doxorubicin cardiotoxicity [67]. Recent report also suggests that targeting HO-1 to mitochondria can prevent inflammation-triggered mitochondrial oxidative stress and apoptosis [68]. Since the side effects of chronic GTN therapy are mainly based on adverse regulation of mitochondrial function such as the inhibition of mitochondrial aldehyde dehydrogenase (ALDH-2) [23], increase in mitochondrial oxidative stress [23, 27], increase in cellular apoptosis [53], the induction of a mitochondrial antioxidant principle (HO-1) would be most effective to prevent GTN-induced tolerance and endothelial dysfunction. This concept was supported by previous reports on the improvement of GTN side effects by a mitochondria-targeted antioxidant (mitoQ) [30], by aggravation of GTN toxicity by partial deficiency in the mitochondrial superoxide dismutase (Mn-SOD) [28], and by interference of blockers of the mitochondrial pores (cyclosporine A and glibenclamide) with the crosstalk between cytosolic and mitochondrial sources of RONS [69]. Vice versa, induction of HO-1 may explain the beneficial profile of PETN [70]. This concept is summarized in Figure 3 and was previously published [31]. To describe the content of this scheme briefly: *In vivo* treatment with PETN is devoid of tolerance and endothelial dysfunction induction in response to chronic *in vivo* treatment. In contrast to GTN, PETN does not increase vascular oxidative stress and therefore does not interfere with its bioactivation by ALDH-2. A likely explanation for this beneficial property of PETN is the induction of the antioxidant enzyme HO-1 and subsequent increases in the expression of ferritin as well as other protective downstream mechanisms not shown here (e.g., ecSOD, GCH-1, and sGC) in vascular tissue but also in the heart. This favorable characteristics of PETN may also explain why therapy with GTN but not PETN causes tolerance and stimulates ROS production in human subjects [9, 71].

## 4. Effects of Organic Nitrates on Gene Expression

There are several examples in the literature showing a transcriptional as well as posttranscriptional modulation of gene expression by nitric oxide (NO) [72–75]. The activity of several transcription factors like NF-*κ*B, AP1 [74], or NRF2 [76] as well as RNA-binding protein like HuR [77] has been shown to be modulated by NO (directly or indirectly via cGMP). Organic nitrates are believed to be indirect NO-donors. Therefore, it seems very likely that treatment with organic nitrates may have implications on the expression of multiple genes. GTN has been described to enhance the expression of c-fos, COX-2, Bcl2, and nNOS in brain nuclei [78–81], to reduce beta-catenin expression in colon cancer cells [82], and to reduce NOX1, NOX2, NOX4 and ALDH2-expression in rat aorta and rat smooth muscle cells [83]. Using microarray analysis, Wang et al. described changes of the expression of 290 genes in the aortas of rats treated with GTN for 8 h [84]. Analyzing the gene expression in the hearts treated for 4 days with GTN, the authors described expressional changes of more than 500 genes [85].

There are also some reports about the expressional effects of PETN [29, 31, 35, 36, 62]. PETN (but not GTN) has been shown to enhance the expression of the antioxidant genes HO-1 and ferritin heavy chain (FeHc) in human endothelial cells [29, 35, 36, 62] and rat aorta [31]. In microarray experiments, the authors showed that PETN modulated the expression of more than 1200 genes in the hearts of rats treated with PETN for 4 days [85].

## 5. Molecular Mechanisms Involved in the Regulation of Gene Expression by Organic Nitrates

**Figure 4:**
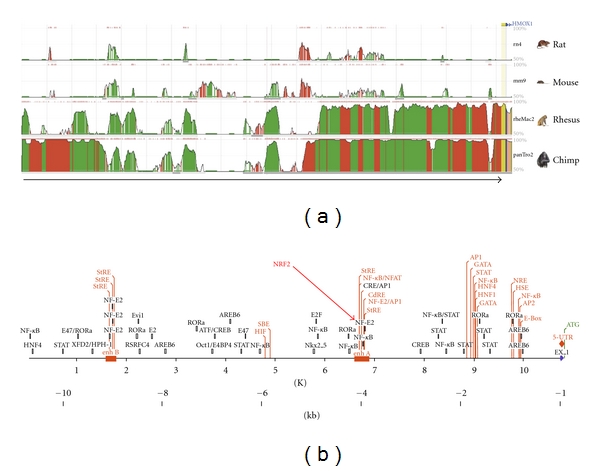
Comparison of the HO-1 promoter sequences of different species. (a) Comparison of the 5′-flanking sequences (10 kb) of the rat, mouse, rhesus macaque, chimpanzee, and human (arrow) HO-1 gene using the ECR-Browser software (http://ecrbrowser.dcode.org/). The search area was 10 bp, and the minimal homology was 80%. The height of the curves (50% < *X* < 100%) indicates the homology (red: intergenic regions, green: single repeats, yellow: untranslated regions of the RNA (UTR), blue: exon, salmon: intron, pink ECR, above). (b) Map of transcription factor binding sites (TFBS) in the human HO-1 promoter. TFBS labeled in red have been verified experimentally in different cells systems (see also [33]).

The comparison of the 5′-flanking sequences of the HO-1 gene (promoter, 10 kb) in different species (rat, mouse, rhesus macaque, chimpanzee, and human) displays regions with very high homology between these species (“evolutionary conserved regions,” ECRs; see Figure 4). These ECR are likely to be involved in the regulation of the HO-1 promoter activity. Bioinformatic analyses show that these ECR contain the binding sites of transcription factors (e.g., NRF2) known to regulate HO-1 promoter activity in different mammalian cell systems (e.g., macrophages, fibroblasts, smooth muscle cells [33]) after various stimuli. In human SH-SY5Y neuroblastoma [86] or rat vascular smooth muscle cells [87], the enhancement of the HO-1 expression by different NO donors DETA-NO has been shown to depend on the expression of NRF2.

**Figure 5:**
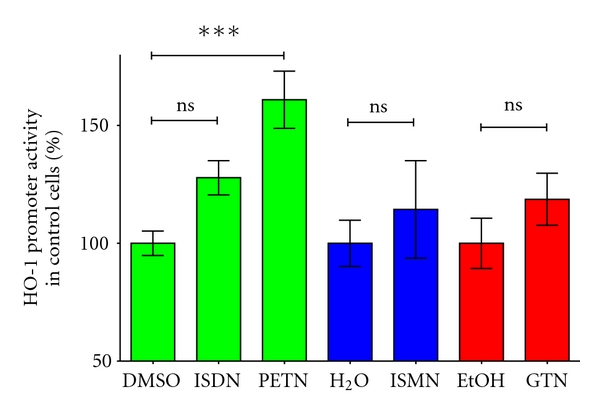
Effects of organic nitrates on the human HO-1 promoter activity. DLD1-HO-11kb-Prom cells were treated for 8 h with isosorbide dinitrate ISDN, PETN, isosorbide-5-mononitrate ISMN, or GTN at a concentration of 50 *μ*M or the respective solvent (DMSO, H_2_O, EtOH). Extracts were prepared, and luciferase activity and protein content were measured, and luciferase activity was normalized to the protein content. Shown (mean ± SEM; *n* = 8–10) are the normalized luciferase activity values. The normalized luciferase activity of the cell treated with solvent was set to 100%. (***= *P* < 0.001, ns: not significant different from solvent-treated cells.) These data were partly published in [34].

To analyze the molecular mechanisms of the regulation of the HO-1 expression by organic nitrates, the authors cloned a 11 kb fragment of the human HO-1 promoter into a luciferase reporter gene construct. This construct was stably transfected into human epithelial DLD-1 cells (DLD1-HO-11kb-Prom). These cells were treated for 8 h with PETN, ISDN, ISMN, or GTN (or the respective solvents, dimethyl sulfoxide DMSO, H_2_O, ethanol EtOH). Cell extracts were prepared, and luciferase activity was measured. As shown in Figure 5, only PETN was able to enhance the promoter activity of the human HO-1 gene. Therefore, the enhancement of HO-1 expression by PETN seems to depend at least partly on PETN-induced enhancement of the HO-1 promoter. A similar comparison of the effects of different organic nitrates on HO-1 induction was recently published [34].

**Figure 6:**
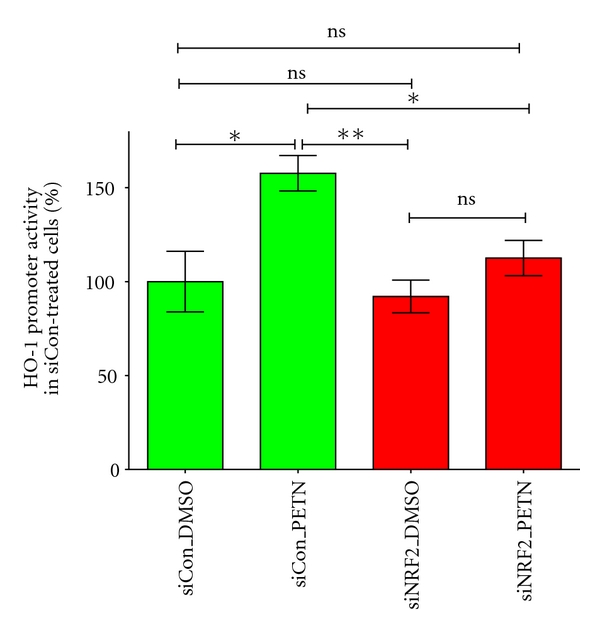
PETN-induced enhancement of the human HO-1 promoter activity depends on NRF2. DLD1-HO-11kb-Prom cells were transfected with a specific anti-NRF2 siRNA (siNRF2) or a nonrelated control siRNA (siCon). After 48 h, the cells were incubated with PETN or the solvent DMSO. Extracts were prepared and luciferase activity and protein content were measured, and luciferase activity was normalized to the protein content. Shown (mean ± SEM; *n* = 5-6) are the normalized luciferase activity values. The normalized luciferase activity of the cell treated with solvent and siCon was set to 100%. (**= *P* < 0.01, *= *P* < 0.05, ns: not significant different to DMSO and siCon-treated cells. Modified from [34].

To analyze the involvement of the transcription factor NRF2 in this PETN-mediated enhancement of HO-1 promoter activity, NRF2-knockdown experiments using a specific anti-NRF2 siRNA were performed. These experiments clearly showed that the PETN-mediated enhancement of the human HO-1 promoter activity depends on the NRF2 expression (see Figure 6) [34]. Therefore, it is likely to speculate that the NO generated by PETN activates NRF2 which in turn binds to the HO-1 promoter and enhances transcription.

**Figure 7:**
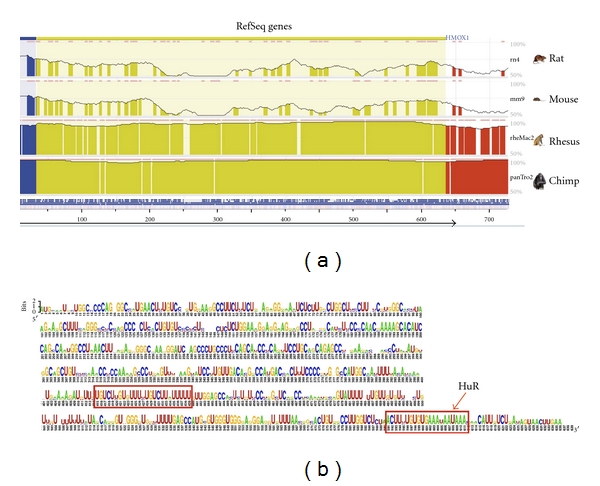
Comparison of the 3′-UTR of the HO-1 mRNA of different species. (a) The 3′-UTR sequences of the HO-1 mRNA from rat, mouse, rhesus macaque, chimpanzee and human was compared using the ECR-Browser software. The search area was 10 bp and the minimal homology was 80%. The height of the curves (50% < *X* < 100%) indicates the homology (red = intergenic regions, yellow = untranslated regions of the RNA (UTR), blue = exon, pink ECR, above). (b) Using the software RNALogo (http://rnalogo.mbc.nctu.edu.tw/createlogo.html) a consensus sequence of all 5 3′-UTR sequences was generated. The height of the letters indicate the frequency of the appearance of this base. AREs are marked by a red box. A putative HuR binding site is indicated.

Beside transcriptional regulation, the modulation of expression of HO-1 has been shown to depend on posttranscriptional mechanisms [77, 88–92]. Posttranscriptional regulation of mRNA stability and translatability mostly depends on sequences found in the 3′-untranslated sequence (3′-UTR) of the mRNAs [93, 94]. Analysis of the 3′-UTR sequences of the HO-1 mRNA of different species (rat, mouse, rhesus macaque, chimpanzee, and human, see Figure 7) reveals evolutionary conserved regions (ECR). In the 3′-UTR sequences of all species, AU-rich elements (AREs) is highly conserved. AREs have been shown to be the binding sites of RNA, binding proteins like HuR or KSRP, which stabilize or destabilize the mRNAs. In a recent paper, the NO-dependent stabilization of the HO-1 mRNA in murine fibroblasts was shown to depend on the RNA-binding protein HuR [77].

**Figure 8:**
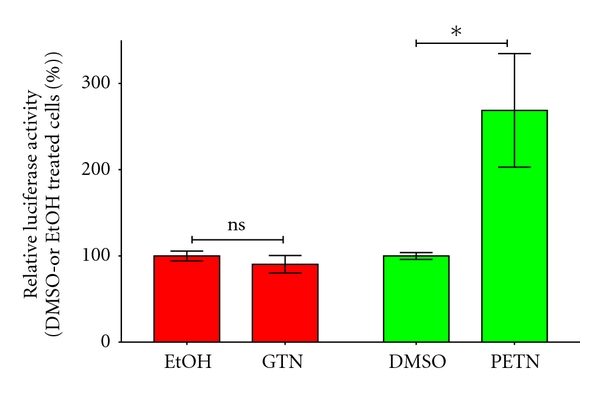
Post-transcriptional regulation of the human HO-1 expression. Human endothelial EA.hy 926 cells were transiently transfected with pGL3-Control-HO-1-3-UTR and pRenilla (normalization of transfection efficiency). After 24h the cells were treated with 50 *μ*M nitroglycerin (GTN) or PETN (or the solvents ethanol [EtOH] or DMSO) for 6h. Extracts were prepared and luciferase and Renilla activity were determined. The luciferase activity was normalized to the renilla activity. Shown (mean ± SEM; *n* = 6–8) are the normalized luciferase activity values. The normalized luciferase activity of the cell treated with solvent were set to 100% (*= *P* < 0.05; ns = not significant versus EtOH- or. DMSO treated cells).

To analyze the effects of organic nitrates on the HO-1 mRNA stability, the authors cloned the 3′-UTR sequence of the human HO-1 mRNA behind the luciferase reporter gene (pGL3-Control-HO-1-3-UTR). Stable human endothelial cells (EA.hy 926) were transiently transfected with this construct and treated with GTN or PETN (see Figure 8). PETN but not GTN enhanced luciferase activity in the transfected cells indicating a PETN-dependent stabilization of the HO-1 mRNA (unpublished data, Hartmut Kleinert).

## 6. Conclusions and Clinical Implications

**Figure 9:**
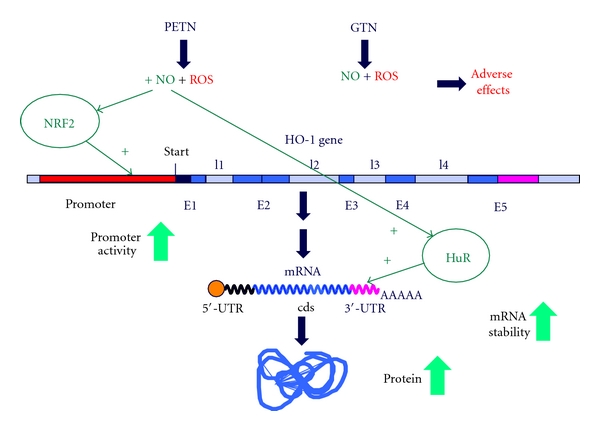
Molecular mechanisms of PETN-mediated enhancement of HO-1 expression. The high amounts of bioactive NO generated from PETN (but not GTN) activate the transcription factor NRF2 and thereby enhance the HO-1 promoter activity. In addition, the interaction of the stabilizing RNA binding protein HuR with the 3′-UTR of the HO-1 mRNA is enhanced. Both effects result in an enhancement of HO-1 expression (E: exon, I: intron).

Organic nitrates like GTN or PETN seem to have marked distinct pharmacological properties and side effects [50], translating to different therapeutic profiles and clinical properties (e.g., induction or lack of nitrate tolerance) [9], which may be at least in part explained by their different effects on HO-1 gene expression [2]. This may be attributed to the different amounts of bioactive NO generated from these compounds. As GTN markedly enhances ROS production in cells, only small amounts of bioactive NO are produced. In contrast, PETN incubation results in decent amount of bioactive NO (or a related species such as S-nitrosothiols or heme-NO) resulting in enhanced NRF2 binding to the HO-1 promoter and HuR binding to the HO-1 mRNA. Thereby, PETN enhances HO-1 expression both by transcriptional and posttranscriptional effects (see Figure 9). Since HO-1 and its products may directly regulate other genes [47], distinct modulation of HO-1 expression by different organic nitrates may also explain differential regulation of gene expression by organic nitrates in general (e.g., GTN versus PETN) [85].

First clinical evidence for the importance of HO-1 to overcome nitrate tolerance is based on the observation that statin therapy (well-known inducers of HO-1) was able to prevent nitrate tolerance in human subjects [40, 41]. Despite the fact that published preclinical data clearly show that an organic nitrate with HO-1 inducing properties such as PETN has less side effects [31] and even has beneficial effects on experimental hypertension [32], diabetes [34], and atherosclerosis [95], these findings still lack molecular proof in human subjects to increase the clinical importance of this concept. A proof of concept study aiming to demonstrate that individuals with Morbus Meulengracht (Gilbert's syndrome), displaying increased HO-1 activity and bilirubin levels, are devoid of tolerance, endothelial dysfunction and oxidative stress in response to nitroglycerin (GTN) therapy could provide a new therapeutic option to overcome these undesired side effects of GTN treatment.

##  Authors' Contribution

A. Daiber and H. Kleinert wrote the first draft, prepared the figures, and designed the original research. M. Oelze, P. Wenzel, F. Bollmann, and A. Pautz commented on subsequent drafts and have performed the original experiments. All authors have approved the paper.
